# Role of COVID-19 Vaccines in SARS-CoV-2 Variants

**DOI:** 10.3389/fimmu.2022.898192

**Published:** 2022-05-20

**Authors:** Zhou Zhou, Yimiao Zhu, Ming Chu

**Affiliations:** ^1^ Department of Immunology, School of Basic Medical Sciences, Peking University, NHC Key Laboratory of Medical Immunology (Peking University), Beijing, China; ^2^ Department of Clinical Pharmacology, Nanjing First Hospital, Nanjing Medical University, Nanjing, China; ^3^ Department of Pharmacy, Shenyang Pharmaceutical University, Shenyang, China

**Keywords:** COVID-19, SARS-CoV-2, Delta variant, Omicron variant, COVID-19 vaccines

## Abstract

Coronavirus disease 2019 (COVID-19), caused by severe acute respiratory syndrome coronavirus-2 (SARS-CoV-2), is a threat to the health of the global population. As the result of a global effort in the determination of origin, structure, and pathogenesis of SARS-CoV-2 and its variants, particularly such the variant of concern as Delta Variant and Omicron Variant, the understanding of SARS-CoV-2 are deepening and the development of vaccines against SARS-CoV-2 are ongoing. Currently, AstraZeneca-Vaxzevria/SII-Covishield vaccine, Janssen-Ad26.COV2.S vaccine, Moderna-mRNA-1273 vaccine, Pfizer BioNTech-Comirnaty vaccine and Sinovac-CoronaVac vaccine have been listed as WHO Emergency Use Listing (EUL) Qualified Vaccines by WHO. Because of the antigen escape caused by the mutation in variants, the effectiveness of vaccines, which are currently the main means of prevention and treatment, has been affected by varying degrees. Herein, we review the current status of mutations of SARS-CoV-2 variants, the different approaches used in the development of COVID-19 vaccines, and COVID-19 vaccine effectiveness against SARS-CoV-2 variants.

## 1 Introduction

Coronavirus disease 2019 (COVID-19), caused by severe acute respiratory syndrome coronavirus-2 (SARS-CoV-2), primarily emerged at the end of December 2019. It has spread rapidly and globally and has caused a disastrous effect, resulting in more than 435 million confirmed infections and 5.9 million deaths as of March 1, 2022 (1). The most common symptoms at the onset of the disease are fever, cough, dyspnea, etc.

Coronavirus is an enveloped virus with a positive single-stranded RNA genome. It can be divided into four genera, α, β, γ, and δ, according to the serotype and genome characteristics (2). SARS-CoV-2 belongs to the β-coronavirus subgenus, having a typical genome structure as other β-coronaviruses. The structural spike (S) glycoprotein, envelope (E) protein, nucleocapsid (N) protein, and membrane (M) glycoprotein are encoded in a specific order within the genome (3). The S glycoprotein could attach to the angiotensin-converting enzyme 2 (ACE2) receptor to enter and infect the targeted cell, thereby blocking the renin–angiotensin–aldosterone pathway, leading to increased levels of angiotensin II and ACE2 levels. This may also be the main reason for the cytokine storm and acute respiratory distress in COVID-19 patients.

Like other RNA viruses, SARS-CoV-2 undergoes a high degree of genomic mutation in the process of adapting to the host. The World Health Organization (WHO) classifies variants into variant of concern (VOC) and variant of interest (VOI) based on their characteristics. VOC has undergone huge malignant changes in transmissibility and virulence that are different from the ancestral strain, which also poses new challenges and limitations to existing treatment options, which poses very large hidden dangers to existing prevention and treatment methods.

In order to effectively prevent the new coronavirus infection and control the spread of the virus, scholars and institutions around the world have conducted research on a wide range of treatment strategies, including vaccine, immunotherapy, and antiviral agents (4). As the best method to prevent and treat COVID-19, the current types of vaccines are inactivated vaccines, viral vector vaccines, DNA vaccines, and mRNA vaccines. According to WHO statistics, a total of 148 candidate vaccines in the world are currently in clinical development, and 195 vaccines are in pre-clinical development (5). Fortunately, more than 10 billion vaccine doses have been administered, enhancing the defense line for human beings.

Although the diagnosis and treatment plan are constantly updated with in-depth research and the treatment methods are becoming increasingly mature, COVID-19 continues to spread globally and has not been completely under control. This reviews the current status of mutations of the new coronavirus and the impact of mutations on treatment. In this review, we hope to provide references for subsequent vaccine development and clinical research.

## 2 The Structure and Genome Organization of SARS-CoV-2

The characterization and determination of the structure and genomic organization of SARS-CoV-2 will help us understand and explain the mechanism of viral infection and mutation, allowing us to choose the available medical treatment.

**Figure 1:**
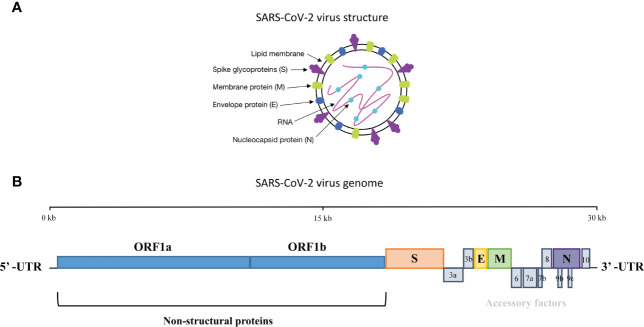
The structure and genome organization of SARS-CoV-2. **(A)** Four structural proteins in SARS-CoV-2 are the spike (S), envelope (E), nucleocapsid (N) and membrane (M). S, E and M are embedded in the lipid bilayer envelope while RNA are coated by N. **(B)** The genome of SARS-CoV-2 consists of 13-15 open reading frames and encodes protein which plays important role in binding and translation between the virus and the host cell.

Coronaviruses are enveloped viruses with positive single-stranded RNA genomes (6). They can be divided into four genera, α, β, γ, and δ, according to the serotype and genome characteristics (2). SARS-CoV-2 belongs to the β-coronavirus subgenus, having a typical genome structure as other β-coronaviruses with ~29 kb RNA genome size. The genome consists of 13–15 open reading frames (ORFs) and begins with a 5’-terminal untranslated region (UTR) and ends with 3’-UTR (7), both of which play an important role in binding and translation between the virus and the host cell. The first ORF (ORF1a/1b), located in 5’-UTR, which contains two-thirds of the genome, encodes the replicase polyprotein 1a (PP1a) and polyprotein 1ab (PP1ab), respectively. The rest of the genome close to 3’-UTR encodes four structural proteins, namely, the spike (S) glycoprotein, the envelope (E) protein, the nucleocapsid (N) protein, and the membrane (M) glycoprotein (4) (**Figure 1**).

The S glycoprotein is a homotrimer located on the 3’-UTR of the SARS-CoV-2 genome and present on the surface of the virus, which forms the unique characteristic of a crown-like shape on the outer surface of the virus under an electron microscope. The S glycoprotein is composed of an N-terminal S1 subunit and C-terminal S2 subunit (8), and plays a vital role in mediating receptor binding and membrane fusion. The S1 subgroup can be divided into four domains: SA, SB, SC, and SD. Unlike MERS-COV that binds to dipeptidyl peptidase-4 (DPP4) receptors through SA and SB domains, SARS-CoV-2 binds to human angiotensin-converting enzyme 2 (hACE-2) receptors through the SB domain. The S2 subunit has three long α-helixes, multiple α-helical segments, extended twisted β-sheets, a membrane-spanning α-helix, and intracellular cysteine-rich fragments, making it similar to a “Stem” structure. The uniqueness that is different from other coronaviruses is that there are O-linked glycans and PRRA protein sequences in the genome of the S glycoprotein of SARS-CoV-2.

In the process of approaching and binding to the receptor, the S glycoprotein will be cleaved twice at two sites. The first cleavage occurs at the polybasic furan-cleavage site existing at the boundary of the S1 and S2 subunits. PRRA is the reason why it can be cleaved effectively by furans and other proteases. This cleavage creates a non-covalent membrane boundary, which enhances the binding affinity of the protein, thereby activating the protein for extensive fusion. The second cleavage occurs at the S2’ cleavage point upstream of the fusion peptide present in the S2 subunit. This cleavage exposes the fusion peptide inserted into the membrane, which promotes membrane fusion and subsequent virus entry into the host cell (9–11).

## 3 The Outline of SARS-CoV-2

As mentioned earlier, SARS-CoV-2 undergoes a high degree of genomic mutation that causes antigenic drift, resulting in an escape from immune recognition in the process of adapting to the host. Compared to the ancestral strain, various variants exhibit specific characteristics. In late 2020, WHO classify variants as variants of interest (VOIs), variants of concern (VOCs), and variants under monitoring (VUMs) based on their characteristics and the risk posed to global public health.

### 3.1 Variant of Concern

The variant can be classified as VOC if it shows one or more of the following characteristics: (a) an increase in transmissibility or harmful changes in COVID-19 epidemiology; (b) an increase in virulence or harmful changes in clinical disease manifestations; and (c) a decrease in the effectiveness of the existing diagnostic, vaccine, and treatment measures (12). So far, there are five variants identified as VOCs: Alpha (B.1.1.7), Beta (B.1.351), Gamma (P.1), Delta (B.1.617.2), and Omicron (B.1.1.529). In short, the transmission, morbidity, and mortality rates of these VOCs have increased dramatically, and the ability of VOCs to evade identification *via* existing diagnostic tests, leading to vaccine breakthrough cases in previously infected individuals, reinfected individuals after recovery, and fully vaccinated individuals, has been developed.

#### 3.1.1 Alpha (B.1.1.7)

The B.1.1.7 variant, known as the Alpha variant or GRY, was discovered in the United Kingdom in September 2020 and was designed on December 18, 2020 (13, 14). It includes 17 mutations in the viral genome. Of these, 9 mutations (H69_V70 deletion, Y144 deletion, N501Y, A570D, D614G, P681H, T716I, S982A, and D1118H) are found in the spike protein. Among the mutations, N501Y’s protruding protein mutation plays an extremely important role in enhancing affinity with hACE-2 receptors and enhancing viral adhesion and the ability to enter host cells. Meanwhile, S-gene target failure (SGTF) is negatively affected by the partial absence of the S gene (15–17) (**Table 1**).

**Table 1:** The summary of variants of concern.

WHO label	Pango lineage	GISAID clade	Earliest documented samples	Date of designation	Number of mutations in Spike protein	Site of mutation in Spike protein	Transmission (increase in reproduction number compared to ancestral strain)	Immune evasion^*^
Alpha	B.1.1.7	GRY	United Kingdom, September 2020	December 18, 2020	9	H69_V70 deletion	29% (95% CI: 24%–33%) (18)	2.3 times (19)
						Y144 deletion		
						N501Y		
						A570D		
						D614G		
						P681H		
						T716I		
						S982A		
						D1118H		
Beta	B.1.351	GH/501Y.V2	South Africa, May 2020	December 18, 2020	8	L242_L244 deletion	25% (95% CI: 20%–30%) (18)	8.2 times (19)
						D80A		
						D215G		
						K417N		
						E484K		
						N501Y		
						D614G		
						A701V		
Gamma	P.1	GR/501Y.V3	Brazil, November 2020	January 11, 2021	12	L18F	38% (95% CI: 29%–48%) (18)	N/A
						T20N		
						P26S		
						D138Y		
						R190S		
						K417T		
						E484K		
						N501Y		
						D614G		
						H655Y		
						T1027I		
						V1176F		
Delta	B.1.617.2	GK	India, October 2020	May 11, 2021	9	E156_F157 deletion	97% (95% CI: 76%–117%) (18)	5.7 times (19)
						T19R		
						G142D		
						R158G		
						L452R		
						T478K		
						D614G		
						P681R		
						D950N		
Omicron	BA.1	GRA	Multiple countries, November 2021	November 26, 2021	34	H69_V70 deletion	N/A	N/A
						V143_Y145 deletion		
						N211 deletion		
						R214_insEPE		
						A67V		
						T95I		
						G142D		
						L212I		
						G339D		
						S371L		
						S373P		
						S375F		
						K417N		
						N440K		
						G446S		
						S477N		
						T478K		
						E484A		
						Q493R		
						G496S		
						Q498R		
						N501Y		
						Y505H		
						T547K		
						D614G		
						H655Y		
						N679K		
						P681H		
						N764K		
						D796Y		
						N856K		
						Q954H		
						N969K		
						L981F		
	BA.1.1				35	H69_V70 deletion		
						V143_Y145 deletion		
						N211 deletion		
						R214_insEPE		
						A67V		
						T95I		
						G142D		
						L212I		
						G339D		
						R346K		
						S371L		
						S373P		
						S375F		
						K417N		
						N440K		
						G446S		
						S477N		
						T478K		
						E484A		
						Q493R		
						G496S		
						Q498R		
						N501Y		
						Y505H		
						T547K		
						D614G		
						H655Y		
						N679K		
						P681H		
						N764K		
						D796Y		
						N856K		
						Q954H		
						N969K		
						L981F		
	BA.2				28	L24_A27delinsS		
						T19I		
						G142D		
						V213G		
						G339D		
						S371F		
						S373P		
						S375F		
						T376A		
						D405N		
						R408S		
						K417N		
						N440K		
						S477N		
						T478K		
						E484A		
						Q493R		
						Q498R		
						N501Y		
						Y505H		
						D614G		
						H655Y		
						N679K		
						P681H		
						N764K		
						D796Y		
						Q954H		
						N969K		
	BA.3				N/A	N/A		

*The study measured the ability of immune evasion of SARS-CoV-2 variants using sera from 12 individuals infected during the first UK wave in mid-2020.

N/A means that no data are available.

According to the previous clinical cohort study conducted in Scotland, Alpha variant infection was positively correlated with an increase in clinical severity compared to non-VOC SARS-CoV-2 infection [cumulative OR 1.40 (95% CI: 1.02–1.93)]. Additionally, the viral load of the Alpha variant, measured by the cycle threshold (Ct) value, was lower than that of non-VOC variants, whereas a lower Ct value indicates a higher viral load (20).

#### 3.1.2 Beta (B.1.351)

The second SARS-CoV-2 variant, B.1.351, is also known as the Beta variant or GH/501Y.V2. In May 2020, this variant was first discovered in South Africa and was designed on December 18, 2020 (21). It includes 8 mutations (L242_L244 deletion, D80A, D215G, K417N, E484K, N501Y, D614G, and A701V) in the spike protein, of which 3 mutations (K417N, E484K, and N501Y) are located in the receptor binding domain (RBD), increasing the binding affinity with hACE-2 receptors, resulting in a higher risk of transmission and reducing monoclonal antibody therapy, convalescent sera, and post-vaccination sera (22, 23). The study from the United Kingdom revealed a significant increase in the pooled mean effective reproduction number relative to the ancestral strain of the Beta variant at 25% (95% CI: 20%–30%) (18) (**Table 1**).

#### 3.1.3 Gamma (P.1)

The third SARS-CoV-2 variant is the P.1 variant, also known as the Gamma variant or GR/501Y. V3 was primarily found in Brazil in November 2020 and was designed on January 11, 2021. It has 12 mutations (L18F, T20N, P26S, D138Y, R190S, K417T, E484K, N501Y, D614G, H655Y, T1027I, and V1176F) in the spike protein. Three mutations (N501Y, K417T, and E484K) are located in the RBD, similar to the B.1.351 variant. According to the study from the United Kingdom, the Gamma variant is about 1.7- to 2.4-fold more transmissible; meanwhile, the infection with P.1 is 1.2 to 1.9 times more likely to cause mortality in the period after the emergence of P.1 (24, 25) (**Table 1**).

#### 3.1.4 Delta (B.1.617.2)

The fourth SARS-CoV-2 variant, B.1.617.2, also referred to as the Delta variant or GK, was first detected in India in October 2020, causing a second wave of deadly COVID-19 infections in India. On April 4, 2021, the Delta variant was originally considered as a VOI. However, the rapid spread of this variant around the world prompted WHO to list it as a VOC on May 11, 2021. The B.1.617.2 mutant contains 9 mutations (E156_F157 deletion, T19R, G142D, R158G, L452R, T478K, D614G, P681R, and D950N) in the spike protein (**Table 1**).

Based on previous research, the results showed larger differences in the amount of viral loads with the Delta variant and other variants: high levels of viral loads (2.5-fold) were observed from Beta, while infections with the Alpha and Delta variants had a similar viral load. Delta has a higher replication ability than Alpha, and the spike protein mutation of P861R on the Furin cleavage site makes the Delta variant easier to replicate and thus has a stronger transmission, whereas a previous systematic review found that the Delta variant’s basic reproduction number of 5.08 is much higher than the average value of non-VOCs (2.79) (26). Multiple-country studies have shown that the Delta variant has a higher infection rate among unvaccinated populations than Alpha. Overall, B.1.617.2 shows characteristics such as increased transmissibility and secondary attack rate that are different from other VOCs, increasing the risk of hospitalization and reducing the neutralizing activity.

#### 3.1.5 Omicron (B.1.1.529)

The current SARS-CoV-2 variant, B.1.1.529, was given the name Omicron or GRA and was designed as a VOC on November 26, 2021. It consists of four lineages, namely, BA.1, BA.1.1, BA.2, and BA.3. With a substantial number of mutations (34 mutations of BA.1, 35 mutations of BA.1.1, and 28 mutations of BA.2), the Omicron variant shows huge potential in immune escape and transmissibility. Six of these mutations (G339D, N440K, S477N, T478K, Q498R, and N501Y) enhance the binding affinity to the human hACE-2 receptor, while seven mutations (K417N, G446S, E484A, Q493R, G496S, Q498R, and N501Y) are associated with a reduction in neutralization (27) (**Table 1**).

According to a Chinese study, the Omicron variant infects and multiplies 70 times faster than the Delta variant and ancestral strain in human bronchus (28), while an analysis of neutralization data revealed that the Omicron variant has a 20-fold reduction in neutralization in unvaccinated, previously infected individuals, or individuals who had received two doses of vaccine (29). Another study demonstrated that the reproductive number of the Omicron variant is higher than that of the Delta variant, showing that the Omicron variant is more contagious (30). However, the results of recent hospitalizations from the United States showed that the Omicron variant was found to be associated with less severe disease compared to Delta (31, 32). The low prevalence of severe disease may be associated with large-scale vaccination.

### 3.2 Variant of Interests

According to the definition of VOIs, the variant has predictable or known genetic changes that affect viral characteristics (e.g., infectiousness, the severity of the disease, immune escape, diagnosis, or treatment escape). It has been identified as causing significant community transmission or multiple COVID-19 cluster cases in multiple countries, and the number of cases has increased over time, relative prevalence has increased, and other significant epidemiological effects have indicated the emerging risks to global public health (33). Overall, the adverse effects of VOIs were lower than those of VOCs; as of October 3, 2021, Lambda (C.37) and Mu (B.1.621) were classified as VOIs.

#### 3.2.1 Lambda (C.37)

The variant C.37, also known as GR/452Q. V1, was first discovered in Peru in August 2020; it has since spread widely in large communities in several South American countries, with increasing prevalence and morbidity. On June 14, 2021, WHO classified the variant as “VOI” and labeled it “Lambda”. Lambda is classified by the spike protein in the protein, including G75V, T76I, R246_G252 deletion, L452Q, F490S, D614G, and T859N. These carrying mutations have brought suspected phenotypic effects, such as potential increased transmissibility or possible resistance to neutralizing antibodies.

#### 3.2.2 Mu (B.1.621)

B.1.621, also called GH, was first identified in Colombia in January 2021. Although the global prevalence of the Mu variant among sequenced cases has declined and is currently below 0.1%, its prevalence in Colombia (39%) and Ecuador (13%) has consistently increased. Therefore, on August 30, 2021, WHO classified it as a “VOI” and labeled it “Mu”, which also includes its descendent Pango lineage B.1.621.1. The Mu variant has a range of mutations (T95I, Y145S, Y146insN, R346K, E484K, N501Y, D614G, P681H, and D950N), which indicates the potential properties of immune escape. According to the preliminary data presented to the Working Group on Viral Evolution, the reduction in neutralization capacity of monoclonal antibody therapy, convalescent sera, and post-vaccination sera is similar to the Beta variant.

### 3.3 Variants Under Monitoring

These variants are defined as a suspected genetic change in the characteristics of the virus and may pose a future health risk. However, evidence of their phenotypic or epidemiological effect is not yet clear and requires enhanced monitoring and repeated assessment pending new evidence. Since research and understanding of the effects of this type of variant may develop rapidly, variants under this category are subject to increase or decrease at any time and are therefore not assigned WHO labels. Nevertheless, the previous VOIs/VOCs are monitored for a long time under this category and will maintain their designated WHO labels (33). Currently, there are three (B.1.1.318, C.1.2, and B.1.640) variants designated as VUMs.

## 4 Introduction of Vaccines

Since the invention of the smallpox vaccine, Chinese vaccines have been the most widely used and most effective means of preventing the disease in the world today; it has not only successfully prevented thousands of deaths but also continued to explore the possibilities of cellular immune response and anti-terrorism response within humanity. As of March 1, 2022, a total of 147 candidate vaccines in the world are in clinical development, and 195 vaccines are in pre-clinical development. However, a large number of vaccines are not the same, and there are three main approaches to design them, namely, the whole-microbe approach, the subunit approach, and the genetic approach (33).

### 4.1 The Whole-Microbe Approach

Typically, the whole-microbe approach uses the whole virus or bacterium to design vaccines.

#### 4.1.1 Inactivated Vaccines

The inactivated vaccine is a chemical or physical, or a two-way method of inactivating or killing the disease-carrying virus or bacterium such as *via* psoralens hydrogen peroxide, gamma irradiation, UV treatment, heat, formaldehyde, and β-propiolactone. It can be produced on a large scale, but special laboratory facilities are necessary to safely grow viruses or bacteria, which take a relatively long time to produce. Several concerns regarding adverse reactions (ARs) of this platform are febrile reaction, the associations between vaccines and autoimmune disorders, and immunization stress-related response (34).

As of March 1, 2022, there are 21 types of candidate vaccines based on the inactivated virus (IV) under clinical development (**Table 2**).

**Table 2:** The summary of candidate vaccines in clinical phase.

Approach	Platform*	The number of candidate vaccines (5)	The percent of candidate vaccines in total
Whole-microbe approach	IV	21	15%
	LAV	2	1%
	VVr	4	3%
	VVnr	21	15%
	VVr + APC	2	1%
	VVnr + APC	1	1%
	BacAg-SpV	1	1%
Subunit approach	VLP	6	4%
	PS	48	33%
Genetic approach	DNA	16	11%
	RNA	24	17%

*IV, inactivated virus; LAV, live-attenuated virus; VVr, viral vector replicating; VVnr, viral vector non-replicating; VVr + APC, VVr + antigen-presenting cell; VVnr + APC, VVnr + antigen-presenting cell; BacAg-SpV, bacterial antigen-spore expression vector; VLP, virus-like particle; PS, protein subunit.

#### 4.1.2 Live-Attenuated Vaccines

The live-attenuated vaccines require approaches that include the cultivation of the virus under adverse conditions, or gene operations such as recombination, deletion mutants, codon deoptimization, recombination, and control of the replication fidelity involved in offsetting innate immunodeficiency or codon deoptimization genes to create weakened versions of living viruses or very similar viruses that limit the replication process. It can trigger an immune response similar to the one observed in natural infections but does not cause disease. It has been successfully applied in measles–mumps–rubella (MMR) vaccines and chickenpox vaccines, as well as shingles vaccines. However, because of vaccine-induced immunomodulation for a short period of time, the live-attenuated vaccines have ARs that can lead to activation or reactivation of diseases due to impaired immunity, such as herpes zoster (35). Additionally, live-attenuated vaccines are often accompanied by problems with genetic instability and residual virulence and are not suitable for people with compromised immune systems.

As of March 1, 2022, there are 2 types of candidate vaccines based on the live-attenuated virus (LAV) under clinical development (**Table 2**).

#### 4.1.3 Viral Vector Vaccines

Viral vector vaccines use modified and safe vector viruses as a platform such as adeno or pox virus to deliver specific sub-parts in which germ of interest into the body, triggering an immune response. Viral vector vaccines can be divided into non-replication vector vaccines and replication vector vaccines. Compared with non-replication vector vaccines entering cells and producing vaccine antigens but being unable to replicate, the replication vector vaccines, in addition to producing pathogen-specific antigens, can also replicate and produce infectious virus vectors, completing a new round of infection, express more antigens, copy, and thereby more strongly stimulate the triggering of immune response after first-time cell infection.

As of March 1, 2022, there are 22 types of candidate vaccines under clinical development, of which 21 are non-replication viral vector (VVnr) as the platform, 4 are replication viral vector (VVr) as the platform, 2 are VVr and antigen-presenting cell (APC) as the platform, and 1 is VVnr and APC as the platform (**Table 2**).

### 4.2 The Subunit Approach

The subunit vaccine is a vaccine that uses only very specific parts, namely, subunits of the virus or bacterium that the immune system needs to identify (subunits are mostly proteins or sugars). Unlike the three kinds of vaccines that we mentioned earlier, it does not use the whole microbe as a platform, nor does it use a safe virus as a vector. Among the subunit vaccines, protein-based vaccines are the more critical vaccines to respond to outbreaks. It can consist not only of specific immunogenic proteins purified by viruses or virally infected cells but also of recombinant proteins or super-molecular structures called viral-like particles (VLPs). VLPs may contain copies of one or more viral proteins assembled into nanoparticles of 10 to 200 nm. They are similar to viruses but have replication defects because they do not carry viral genetic material. As a result, they are considered safer than whole-microbe vaccines, and their main risks may be caused by the adjuvant, which enhances their immunogenicity.

As of March 1, 2022, there are 48 types of candidate vaccines based on protein subunit (PS) under clinical development and 6 types of candidate vaccines based on VLPs under clinical development (**Table 2**).

### 4.3 The Genetic Approach

Unlike vaccines that use the whole microbe or subunits, nucleic acid-based vaccines use DNA or RNA as the platform to express immunogenic viral proteins. DNA vaccines are based on plasmid DNA to carry pathogen genes. When human cells are infected, DNA is first converted into mRNA, which is then used as a groundwork to create specific proteins. RNA vaccines, which are manufactured based on mRNA or self-replicating RNA, do not need to be transposed to the nucleus to transcribe mRNA. However, given the fact that mRNA is not very stable, they are usually synthesized with modified nucleosides to prevent degradation and use carrier molecular to transport, such as lipid nanoparticles (LNPs), to allow RNA to enter cells. Nanotechnology has played a significant role in the success of some mRNA-based vaccines, such as BNT162b2 (Pfizer-BioNTech) and mRNA-1273 (Moderna). LNP is a special liposome material that improves the encapsulation rate of nucleic acids, facilitates cell penetration, and increases the stability of delivery (36). In general, nucleic acid vaccines send a specific set of instructions to our cells in the form of DNA or mRNA, allowing them to produce specific proteins to stimulate the immune system to recognize and respond. Like the subunit approach, the main potential risks of nucleic acid vaccines are brought by carrier molecules. Although nucleic acid vaccines can be produced on a large scale, nucleic acid vaccines are a new and developing strategy and there are few application precedents before the COVID-19 outbreak. Currently, some studies have shown that the use of PEG complexed with lipids can positively reduce the allergic reactions of vaccines, which contributes to the advancement of vaccine research and development in the future (37).

As of March 1, 2022, 16 types of candidate vaccines based on DNA are under clinical development and 25 types of candidate vaccines based on RNA are under clinical development (**Table 2**).

## 5 COVID-19 Vaccine Effectiveness Against Variants

**Figure 2:**
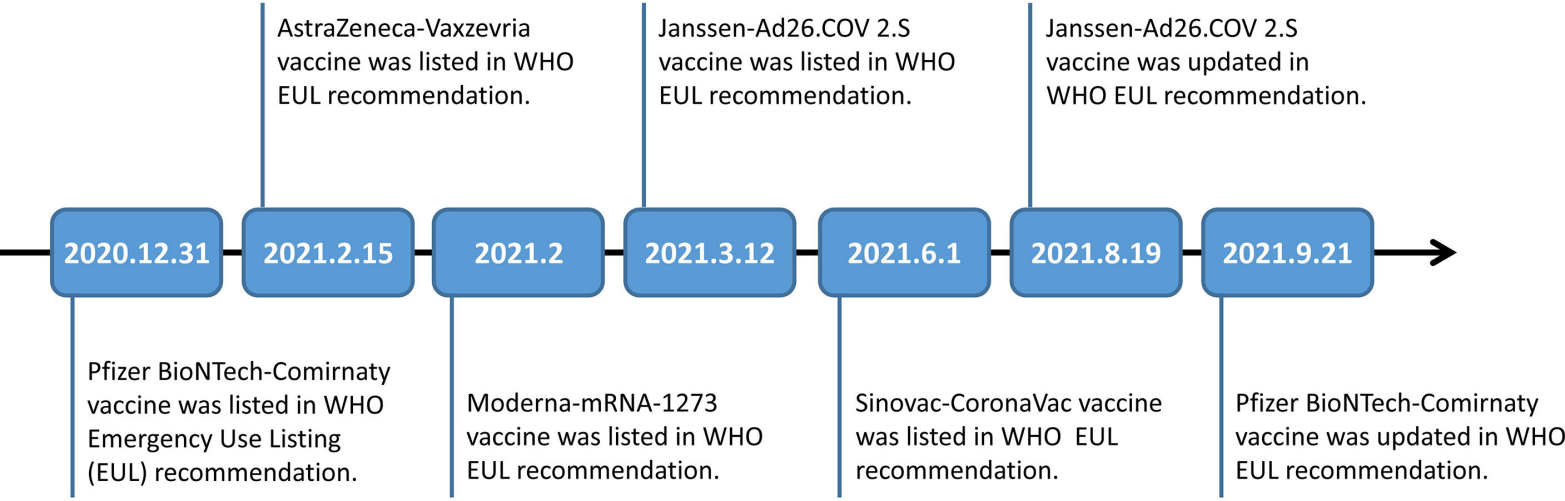
Timeline of Pfizer BioNTech-Comirnaty vaccine, AstraZeneca-Vaxzevria vaccine, Moderna-mRNA-1273 vaccine, Janssen-Ad26.COV 2.S vaccine and Sinovac-CoronaVac vaccine listed in WHO EUL recommendation.

The global spread of variants of COVID-19 has raised widespread public concern about vaccine efficacy (VE) against the variants. So far, AstraZeneca-Vaxzevria/SII-Covishield vaccine, Janssen-Ad26.COV 2.S vaccine, Moderna-mRNA-1273 vaccine, Moderna-mRNA-1273 vaccine/Pfizer BioNTech-Comirnaty vaccine, Pfizer BioNTech-Comirnaty vaccine, and Sinovac-CoronaVac vaccine were categorized under WHO Emergency Use Listing (EUL) Qualified Vaccines (**Figure 2**).

### 5.1 AstraZeneca-Vaxzevria/SII-Covishield Vaccine

AstraZeneca-Vaxzevria vaccine (AZD1222) is a recombinant vaccine, formerly known as ChAdOx1 nCoV-19, developed jointly by Oxford University and AstraZeneca. SII-Covishield vaccine is an AstraZeneca-Vaxzevria vaccine produced in India by SII, licensed by AstraZeneca. The vaccine is a typical example of VVnr vaccines, which use non-replicated chimpanzee adenoviruses to transmit SARS-CoV-2 spike proteins to induce an immune response (38). Pre-clinical studies have shown that the vaccine can induce rapid immune responses to SARS-CoV-2 mediated by type 1 and type 2 T-assisted cells in mice and rhesus monkeys. According to a mid-term analysis of an ongoing multicenter randomized controlled trial, the efficacy of a full dose of the vaccine was found to be 70.4% (95% CI: 54.8–80.6) for symptomatic COVID-19. Additionally, three cases of transverse myelitis among 11,636 participants were reported in this trial. Researchers speculate that one case is likely related to vaccination (39). ARs such as fatigue, headache, fever, and myalgia were also reported in the registration studies for the AstraZeneca-Vaxzevria vaccine (40).

#### 5.1.1 Alpha (B.1.1.7)

A peer-reviewed study from Spain demonstrated that the AZD1222 had 38% (95% CI: −42–73) effectiveness in preventing infection among close contacts against Alpha (B.1.1.7) ≥14 days following the second dose. However, experimental results of those affected by Alpha (B.1.1.7) were hampered by the lack of a large sample of individuals infected with the Alpha variant (41). Another study from the United Kingdom assessed the VE of AZD1222 against documented infection for the Scottish population to be 73% (95% CI: 66–78) (42) (**Table 3**).

**Table 3:** COVID-19 VE^*^ against SARS-CoV-2 variant infection.

COVID-19 Vaccines	Dose	VE % (95% CI) against SARS-CoV-2 Variant Documented Infection				
		Alpha	Beta	Gamma	Delta	Omicron
**United States of America**						
AstraZeneca-Vaxzevria/SII-Covishield vaccine	2nd					
Janssen-Ad26.COV 2.S vaccine	1st	79 (77–80) (43)			78 (73–82) (43)	
Moderna-mRNA-1273 vaccine	2nd	98.4 (96.9–99.1) (44)		95.5 (90.9–97.8) (44)	66.7 (58.9–73.0) (45)	13.9 (10.5–17.1) (46)
Pfizer BioNTech-Comirnaty vaccine	2nd	66.5 (58.3–73.1) (48)			68.9 (61.9–74.7) (45)	25 (20–30) (49)
Sinovac-CoronaVac vaccine						
**Canada**						
AstraZeneca-Vaxzevria/SII-Covishield vaccine	2nd	74 (29–90) (50)	§100 (no CI provided) (51)	90 (61–98) (50)	70 (66–73) (50)	
Janssen-Ad26.COV 2.S vaccine						
Moderna-mRNA-1273 vaccine	2nd	95 (85–98) (50)	§100 (no CI provided) (51)	95 (85–99) (50)	92 (91–93) (50)	
Pfizer BioNTech-Comirnaty vaccine	2nd	96 (93–98) (50)	§81 (−38–97) (51)	93 (89–95) (50)	91 (91–92) (50)	
Sinovac-CoronaVac vaccine						
**Spain**						
AstraZeneca-Vaxzevria/SII-Covishield vaccine	2nd	38 (−42–73) (41)			55 (39–67) (41)	
Janssen-Ad26.COV 2.S vaccine	1st	77 (27–93) (41)			42 (18–59) (41)	
Moderna-mRNA-1273 vaccine	2nd	86 (56–95) (41)			77 (64–85) (41)	
Pfizer BioNTech-Comirnaty vaccine	2nd	71 (61–78) (41)			67 (59–74) (41)	
Sinovac-CoronaVac vaccine						
**United Kingdom**						
AstraZeneca-Vaxzevria/SII-Covishield vaccine	2nd	73 (66–78) (42)			78.9 (66.6–86.7) (27)	11.4 (−18.8–34.6) (27)
Janssen-Ad26.COV 2.S vaccine						
Moderna-mRNA-1273 vaccine	2nd				87.8 (79.8–92.7) (27)	23.7 (4.4–39.4) (27)
Pfizer BioNTech-Comirnaty vaccine	2nd	92 (90–93) (42)			83.5 (78.6–87.3) (27)	26.0 (−18.8–34.6) (27)
Sinovac-CoronaVac vaccine						
**Qatar**						
AstraZeneca-Vaxzevria/SII-Covishield vaccine						
Janssen-Ad26.COV 2.S vaccine						
Moderna-mRNA-1273 vaccine	2nd	100 (91.8–100) (52)	96.4 (91.9–98.7) (52)		73.1 (67.5–77.8) (53)	⊙52 (45.8–57.4) (54)
Pfizer BioNTech-Comirnaty vaccine	2nd	87 (82–91) (55)	72 (66–77) (55)		51.9 (47.0–56.4) (53)	⊙55.5 (51.8–59) (54)
Sinovac-CoronaVac vaccine						
**Brazil**						
AstraZeneca-Vaxzevria/SII-Covishield vaccine	2nd			88.1 (82.8–91.7) (56)	59 (33.1–74.8) (57)	
Janssen-Ad26.COV 2.S vaccine						
Moderna-mRNA-1273 vaccine						
Pfizer BioNTech-Comirnaty vaccine						
Sinovac-CoronaVac vaccine	2nd			46.8 (38.7–53.8) (47)	#55 (54.3–55.7) (58)	
**Czech Republic**						
AstraZeneca-Vaxzevria/SII-Covishield vaccine						
Janssen-Ad26.COV 2.S vaccine					60 (57–63) (59)	47 (45–49) (59)
Moderna-mRNA-1273 vaccine	2nd				71 (65–76) (59)	48 (44–52) (59)
Pfizer BioNTech-Comirnaty vaccine	2nd	96.2 (91.6–98.7) (60)			65 (<0–96.6) (60)	49 (48–50) (59)
Sinovac-CoronaVac vaccine						

*VE refers to vaccine effectiveness.

^#^The VE of Sinovac-CoronaVac vaccine is assessed against symptomatic infection in Brazil.

^§^The VE of vaccine is assessed against symptomatic infection ≥7 days after the 2nd dose.

^⊙^The VE of vaccine is assessed against symptomatic infection among individuals who infected previously after ≥14 days after 2nd dose.

#### 5.1.2 Beta (B.1.351)

The results of the clinical trial revealed that the VE of a double-dose AZD1222 regimen was 70.4% (95% CI: 43.6–84.5) against symptomatic infection for the Beta variant (B.1.351) (61).

#### 5.1.3 Gamma (P.1)

The negative test case–control study from Canada showed that AZD1222 against documented infection for Gamma was surprisingly effective (50). However, the study from Brazil revealed that the VE of double-dose AstraZeneca-Vaxzevria vaccine was 88.1% (95% CI: 82.8–91.7) (56) (**Table 3**).

#### 5.1.4 Delta (B.1.617.2)

Peer-reviewed studies from Spain also demonstrated that AZD1222 was 55% (95% CI: 39–67) effective against documented infection for the Delta variant (41). The results of the negative test case–control study from Canada revealed that the VE against hospitalization due to Delta was >90% ≥14 days following the 2nd dose (50). Furthermore, the VE of AZD1222 against death caused by the Delta variant was 91% (95% CI: 86–94) in the evaluation of the VE of AZD1222 preventing death among 380,532 residents in British Columbia including 27,439 cases ≥14 days following the second dose (62) (**Table 3**).

#### 5.1.5 Omicron (B.1.1.529)

In the negative test case–control study from Scotland, two doses of AZD1222 were 11.44% (95% CI: −18.8–34.6) effective against documented infection for the Omicron variant (27). The retrospective cohort study from the Czech Republic revealed that the VE of AZD1222 was 51% (95% CI: 23–69) against documented infection for the Omicron variant (59) (**Table 3**).

#### 5.1.6 Mu (B.1.621)

According to a retrospective cohort study from Colombia, the VE of AZD1222 against hospitalization and death for the Mu variant among individuals ≥60 years old was 75.4% (95% CI: 48.2–88.3) and 96.3% (95% CI: 88.4–98.8), respectively (63).

### 5.2 Janssen-Ad26.COV 2.S Vaccine

The Janssen-Ad.26.COV2. S vaccine, developed by the Janssen Pharmaceutical Companies of Johnson & Johnson, is a recombinant adenoviral vector vaccine. It is a classic example of VVnr vaccines, which induced powerful neutralizing antibody responses and provided almost complete protection following SARS-CoV-2 infection (64). The Phase III clinical trial evaluated the efficacy of one dose of Ad26.COV2. S vaccine to be 76.7% (95% CI: 54.6–89.1) against severe-critical COVID-19 after administration following ≥14 days (65). According to the documents from the Food and Drug Administration (FDA), the proportion of participants reporting any local ARs and any systemic ARs within 7 days after vaccination were 50.2% and 55.1% among 3,356 participants in the vaccine group, compared with 19.4% and 35.1% of the 3,380 participants in the placebo group. Local ARs primarily include injection site pain (vaccine versus placebo: 48.6% versus 16.7%), erythema (7.3% versus 3.9%), and swelling (5.3% versus 1.6%), while systemic ARs includes headache (38.9% versus 23.7%), fatigue (38.2% versus 21.5%), myalgia (33.2% versus 12.7%), nausea (14.2% versus 9.7%), and fever (9.0% versus 0.6%). The median duration of local ARs and systemic ARs were both 2 days of vaccination. FDA assessed several ARs within 28 days of vaccination, such as embolism, thrombosis, arthritis, and peripheral neuropathy, which were likely potentially associated with the vaccine (66).

#### 5.2.1 Alpha (B.1.1.7)

A peer-reviewed study from Spain also demonstrated that the VE of Janssen-Ad26.COV2.S vaccine was evaluated to be 77% (95% CI: 27–93) against infection among close contacts with Alpha-infected individuals (41) (**Table 3**).

#### 5.2.2 Beta (B.1.351)

One dose of the Janssen-Ad26.COV2.S vaccine provides consistent protection against COVID-19 across various countries during a period when the Beta variant was predominant (67).

#### 5.2.3 Gamma (P.1)

The VE of Janssen-Ad26.COV2.S vaccine against symptomatic disease, hospitalization, and death due to Gamma in Brazil was 50.9% (95% CI: 35.5–63.0), 72.9% (95% CI: 35.1–91.1), and 90.5% (95% CI: 31.5–99.6), respectively (68).

#### 5.2.4 Delta (B.1.617.2)

A peer-reviewed study from Spain also demonstrated that the VE of Janssen-Ad26.COV2.S vaccine against infection for the Delta variant was 42% (95% CI: 18–59), which is lower than that of the Alpha variant (41). Meanwhile, the study from the Czech Republic showed that the Janssen-Ad.26.COV2.S vaccine was 60% (95% CI: 57–63) effective against documented infection for the Omicron variant ≥14 days following the second dose (59).

#### 5.2.5 Omicron (B.1.1.529)

The results of the negative test case–control study from South Africa demonstrated that the booster dose of the Janssen-Ad.26.COV2.S vaccine was 63% (95% CI: 31–81) effective against hospitalization for the Omicron variant after 0–13 days (49). According to the study from the Czech Republic, the VE of two doses of the Janssen-Ad.26.COV2.S vaccine was 47% (95% CI: 45–49) against documented infection for the Omicron variant after ≥14 days (59) (**Table 3**).

#### 5.2.6 Mu (B.1.621)

A retrospective cohort study from Colombia evaluated that the Janssen-Ad.26.COV2.S vaccine was 80% (95% CI: 19.9–95.0) and 75.0% (95% CI: 0.0–93.8) effective against hospitalization and death for the Mu variant, respectively (63).

### 5.3 Moderna-mRNA-1273 Vaccine

The Moderna-mRNA-1273 vaccine, developed by Moderna, is an example of an RNA-based vaccine designed by using the host mechanism to express specific antigens after delivering a genetic sequence into a host cell. In this vaccine, the mRNA template is transported by synthetic LNPs, and the target is the spike protein that elicits an immune response. The results of a phase III clinical trial demonstrated that two doses of the Moderna-mRNA-1273 vaccine was 94.1% (95% CI: 89.3–96.8) effective against COVID-19 illness (69). A case of Bell’s palsy was reported 32 days after the vaccination in this trial (69). Moreover, the interim results showed that one participant experienced transient urticaria, which was assumed to be related to the first dose (70). The Moderna-mRNA-1273 vaccine has common adverse effects such as pain or swelling at the injection site, headache, nausea, vomiting, muscle ache, joint aches and stiffness, tiredness, chills, and fever, while severe allergic reactions are rare, such as difficulty breathing, swelling of the face and throat, fast heartbeat, bodily rash, dizziness, and weakness (71).

#### 5.3.1 Alpha (B.1.1.7)

A peer-reviewed study from Spain also demonstrated that the VE of the Moderna-mRNA-1273 vaccine was found to be 86% (95% CI: 56–95) against infection among close contacts with Alpha-infected individuals (41). Moreover, the result of a negative test but not yet peer-reviewed case–control study demonstrated that the VE of Moderna-mRNA-1273 vaccine against infection ≥14 days following the second dose was 98.4% (95% CI: 96.9–99.1) for the Alpha variant (44) (**Table 3**).

#### 5.3.2 Beta (B.1.351)

One study showed that the vaccine provided continuous protection against infection for the Beta variant (72).

#### 5.3.3 Gamma (P.1)

The results of the study showed that the VE against infection ≥14 days following the second dose was 95.5% (95% CI: 90.9–97.8) for the Gamma variant (44).

#### 5.3.4 Delta (B.1.617.2)

The results of a negative test case–control study demonstrated that the VE of the Moderna-mRNA-1273 vaccine was found to be highly effective against infection for the Delta variant 14 to 60 days following the second dose (VE: 94.1%, 95% CI: 90.5–96.3), while it declined to 80.0% (95% CI: 70.2–86.6) 151–180 days following the second dose (44). Similarly, a retrospective cohort study from Canada found that two doses of the Moderna-mRNA-1273 vaccine against infection and hospitalization was 92% (95% CI: 91–93) and 97% (95% CI: 96–98) effective, respectively, among individuals aged ≥18 years old in British Columbia (50). The VE of the third booster vaccine is assessed to be 86.5% (95% CI: 84.8–88.0) (27) (**Table 3**).

#### 5.3.5 Omicron (B.1.1.529)

According to the results of the negative test case–control study from Scotland, two doses of the Moderna-mRNA-1273 vaccine showed 23.7% (95% CI: 4.4–39.4) effectivity in preventing documented infection, while the third booster showed 46.3% (95% CI: 41.3–51.03) effectivity (27). The VE of two doses of the Moderna-mRNA-1273 vaccine was assessed to be 47% (95% CI: 45–49) against documented infection for the Omicron variant based on the retrospective cohort study from the Czech Republic (59) (**Table 3**).

#### 5.3.6 Mu (B.1.621)

The VE estimates against infection ≥14 days following the second dose was 90.4% (95% CI: 73.9–96.5) for the Mu variant (44).

### 5.4 Pfizer BioNTech-Comirnaty Vaccine

The Pfizer BioNTech-Comirnaty vaccine, developed by Pfizer and BioNTech, is another typical example of RNA-based vaccines. The VE of the Pfizer BioNTech-Comirnaty vaccine against symptomatic infection was 95% (73). A peer-reviewed and large retrospective study from Israel showed that the VE of the Pfizer BioNTech-Comirnaty vaccine against infection, symptomatic disease, severe disease, and deaths after seven or more days following the booster dose was 88% (95% CI: 87–90), 91% (95% CI: 89–92), 92% (95% CI: 82–97), and 81% (95% CI: 59–97), respectively (74). Similar to the Moderna-mRNA-1273 vaccine, its side effects were mainly manifested as local ARs such as mild or moderate injection site pain, and systemic ARs such as headache and fatigue.

#### 5.4.1 Alpha (B.1.1.7)

The VE of the Pfizer BioNTech-Comirnaty vaccine against documented infection for the Alpha variant was 87% (95% CI: 81.8–90.7) based on a retrospective cohort study from Qatar (55). Similarly, a study from the United Kingdom found that Pfizer BioNTech-Comirnaty was 85% (95% CI: 79–89) effective against infection for the Alpha variant among household contacts of Alpha-infected cases ≥14 days following the second dose (75) (**Table 3**).

#### 5.4.2 Beta (B.1.351)

A retrospective cohort study in the population of Qatar found that the VE of the Pfizer BioNTech-Comirnaty vaccine was 75.0% (95% CI: 70.5–78.9) against documented infection for the Beta variant (55) (**Table 3**).

#### 5.4.3 Gamma (P.1)

An observational cohort study in Qatar showed that the analysis of 20 serum samples from 15 participants from the Pfizer BioNTech-Comirnaty vaccine clinical efficacy trial efficiently neutralized the Alpha and Gamma variants equally (76).

#### 5.4.4 Delta (B.1.617.2)

A peer-reviewed study from Spain also demonstrated that the VE of the Pfizer BioNTech-Comirnaty vaccine was found to be 67% (95% CI: 59–74) effective against documented infection for the Delta variant (41). However, a study from the United Kingdom found that the Pfizer BioNTech-Comirnaty vaccine had 90% (95% CI: 83–94) effectivity against infection for Delta ≥14 days following the second dose (62). The VE of the booster vaccine was assessed to be 85.9% (95% CI: 84.2–87.4) (27) (**Table 3**).

#### 5.4.5 Omicron (B.1.1.529)

A study from the United States revealed that the Pfizer BioNTech-Comirnaty vaccine was 25% (95% CI: 20–30) effective against documented infection ≥14 days following the second dose (77). The retrospective cohort study from Israel indicated that the booster vaccine was 30% (95% CI: −9–55) effective against infection among healthcare workers who completed administration with three doses at least 4 months previously (78). Based on the study from Qatar, two doses of the Pfizer BioNTech-Comirnaty vaccine against symptomatic infection due to the Omicron BA.1 variant and BA.2 variant among individuals who were infected previously were assessed to be 51.7% (95% CI: 43.5–58.7) and 55.1% (95% CI: 50.9–58.9) effective, respectively (54) (**Table 3**).

#### 5.4.6 Mu (B.1.621)

Based on the retrospective cohort study from Colombia, the Pfizer BioNTech-Comirnaty vaccine against hospitalization and death due to the Mu variant was assessed to be 90.3% (95% CI: 87.1–92.7) and 98.5% (95% CI: 97.8–98.9) effective, respectively (63).

### 5.5 Sinovac-CoronaVac Vaccine

The Sinovac-CoronaVac vaccine, developed by Sinovac Research and Development Co., Ltd., is a typical sample of IV vaccines. This type of vaccine is generally administered intramuscularly and requires an adjuvant to trigger an immune response, eliciting an immune response directed to different virus proteins, and the whole virus is being used to challenge the immune system. According to the results of a phase I/II clinical trial, most side effects, such as injection site pain, were mild and participants recovered within 48 h. Additionally, the occurrence of fever after vaccination with Sinovac-CoronaVac vaccine was lower than other COVID-19 vaccines. There was one case of urticaria 48 h after the first dose, which was considered to be possibly related to vaccination during phase I. Vaccine-related serious adverse events were not reported in subsequent doses (47).

A negative test case–control study from Brazil found that two doses of the Sinovac-CoronaVac vaccine was 46.8% (95% CI: 38.7–53.8) and 55.5% (95% CI: 46.5–62.9) effective against symptomatic COVID-19 and hospitalization, respectively, for the Gamma variant ≥14 days following the second dose (79).

A retrospective cohort study conducted in Colombia, during a period when the Mu variant was predominant, revealed that the Sinovac-CoronaVac vaccine was 67.2% (95% CI: 63.7–70.4) effective at preventing hospitalization and 77.1% (95% CI: 75.5–78.6) effective at preventing death (63).

Based on a retrospective cohort study from Chile, the VE of the Sinovac-CoronaVac vaccine was 37.9% (95% CI: 36.1–39.6) against documented infection for the Omicron variant among children aged 3–5 years ≥14 days following the second dose (80).

## 6 Conclusion

As we mentioned above, with COVID-19 spreading globally, SARS-CoV-2 undergoes a high degree of genomic mutation that causes antigenic drift resulting in an escape from immune recognition in the process of adapting to the host. The variant “Deltacron”, whose existence was confirmed by French researchers, has a potentially dramatic increase in transmission due to its various mutation sites, raising widespread public concerns (81). In the continuously developing situation where new variants are becoming predominant, people are taking positive and effective measures to deal with this phenomenon. All countries play a great role in vaccine research and development, and there are a variety of vaccines that have been listed through clinical trials. From the current epidemic prevention situation, with the popularization of vaccination, the spread of SARS-CoV-2 will certainly be controlled in the future. As of the end of February 2022, more than 3 billion doses of COVID-19 vaccines had been administrated in China; with a vaccination rate of nearly 90%, China has made impressive achievements in epidemic prevention and control. We call on people around the world to be actively vaccinated, and vaccines should be equally available to all regardless of race, color, age, religion, wealth, and nationality.

The effectiveness of all current vaccines against variants, especially the Omicron variant, has been significantly reduced. Since the whole-microbe approach of using a complete virus as a platform has more targets than the subunit approach or genetic approach, we hypothesized that vaccines developed with the whole-microbe approach will provide more specific and effective protection than vaccines based on the subunit approach or genetic approach when new variants emerge.

All vaccines currently injected have varying degrees of side effects, including fever, headache, fatigue, muscle pain and joint pain, and even allergic reactions. In addition, in some underdeveloped countries and regions, vaccination has not been promoted, and the preventive effect of vaccine has not reached the expected goal. This shows that the technology and process of vaccine research and development still have room for optimization and improvement, including the use of new technologies such as nanotechnology in the delivery of vaccines and the control of harmful substances and temperature in the production process. In the future, researchers from different countries will be able to design vaccines against specific variants with different mutation sites, using more effective and scientific techniques to provide sustained protection. By addressing these challenges, we firmly believe that COVID-19 will be defeated by mankind eventually.

## Author Contributions

MC contributed to conception and design of the study. ZZ and YZ organized the database. ZZ and YZ wrote all versions of the manuscript and tables. ZZ and YZ have contributed equally to this work. All authors contributed to manuscript revision, read, and approved the submitted version.

## Funding

This work was supported by Boehringer Ingelheim, the COVID-19 emergency program from Jinhua in China (2020XG-26), National Natural Science Foundation of China (81603119) and Natural Science Foundation of Beijing Municipality (7174316).

## Conflict of Interest

The authors declare that the research was conducted in the absence of any commercial or financial relationships that could be construed as a potential conflict of interest.

## Publisher’s Note

All claims expressed in this article are solely those of the authors and do not necessarily represent those of their affiliated organizations, or those of the publisher, the editors and the reviewers. Any product that may be evaluated in this article, or claim that may be made by its manufacturer, is not guaranteed or endorsed by the publisher.
